# Synthetic cannabinoid JWH-073 alters both acute behavior and *in vivo/vitro* electrophysiological responses in mice

**DOI:** 10.3389/fpsyt.2022.953909

**Published:** 2022-10-21

**Authors:** Mario Barbieri, Micaela Tirri, Sabrine Bilel, Raffaella Arfè, Giorgia Corli, Beatrice Marchetti, Lorenzo Caruso, Marie Soukupova, Virginia Cristofori, Giovanni Serpelloni, Matteo Marti

**Affiliations:** ^1^Department of Neuroscience and Rehabilitation, University of Ferrara, Ferrara, Italy; ^2^Department of Translational Medicine, Section of Legal Medicine and Laboratory for Technologies of Advanced Therapies (LTTA) Centre, University of Ferrara, Ferrara, Italy; ^3^Department of Environment and Prevention Sciences, University of Ferrara, Ferrara, Italy; ^4^Department of Chemistry and Pharmaceutical Sciences, University of Ferrara, Ferrara, Italy; ^5^Neuroscience Clinical Center and Transcranial Magnetic Stimulation (TMS) Unit, Verona, Italy; ^6^Department for Anti-Drug Policies, Collaborative Center of the National Early Warning System, Presidency of the Council of Ministers, Rome, Italy

**Keywords:** JWH-073, JWH-018, synthetic cannabinoids, NOR, hippocampus, cortex, electrophysiology, NPS

## Abstract

JWH-073 is a synthetic cannabinoid (SCB) that is illegally marketed within an “herbal blend”, causing psychoactive effects more intense than those produced by Cannabis. Users report that JWH-073 causes less harmful effects than other SCBs, misrepresenting it as a “safe JWH-018 alternative”, which in turn prompts its recreational use. The present study is aimed to investigate the *in vivo* pharmacological activity on physiological and neurobehavioral parameters in male CD-1 mice after acute 1 mg/kg JWH-073 administration. To this aim we investigate its effect on sensorimotor (visual, acoustic, and tactile), motor (spontaneous motor activity and catalepsy), and memory functions (novel object recognition; NOR) in mice coupling behavioral and EEG data. Moreover, to clarify how memory function is affected by JWH-073, we performed *in vitro* electrophysiological studies in hippocampal preparations using a Long-Term Potentiation (LTP) stimulation paradigm. We demonstrated that acute administration of JWH-073 transiently decreased motor activity for up to 25 min and visual sensorimotor responses for up to 105 min, with the highest effects at 25 min (~48 and ~38%, respectively), while the memory function was altered up to 24 h (~33%) in treated-mice as compared to the vehicle. EEG in the somatosensory cortex showed a maximal decrease of α (~23%) and γ (~26%) bands at 15 min, β (~26%) band at 25 min, a maximal increase of θ (~14%) band at 25 min and δ (~35%) band at 2 h, and a significant decrease of θ (~18%), α (~26%), and β (~10%) bands during 24 h. On the other hand, EEG in the hippocampus showed a significant decrease of all bands from 10 min to 2 h, with the maximal effect at 30 min for θ (~34%) and γ (~26%) bands and 2 h for α (~36%), β (~29%), and δ (~15%) bands. Notably, the δ band significant increase both at 5 min (~12%) and 24 h (~19%). Moreover, *in vitro* results support cognitive function impairment (~60% of decrease) by interfering with hippocampal synaptic transmission and LTP generation. Our results suggest that JWH-073 deeply alters brain electrical responsiveness with minor behavioral symptoms. Thus, it poses a subtle threat to consumers who mistakenly consider it safer than other SCBs.

## Introduction

JWH-073 [1-butyl-3-(1-naphthoyl) indole] is a synthetic cannabinoid (SCB), a member of the large family of JWH naphthoylindoles compounds. JWH-073 is a computational melding of the chemical structural features of Δ^9^-tetrahydrocannabinol (Δ^9^-THC) with the prototypic aminoalkylindole WIN 55,212-2 developed in the early 1990s. JWH-073 is structurally similar to JWH-018 (1), and like Δ^9^-THC, it has a high binding affinity toward CB_1_ (Ki ranging from 8.9 ± 1.8 and 12.9 ± 3.4 nM) and CB_2_ receptors (38 ± 24 nM) (2–5). Different from Δ^9^-THC, its monohydroxylated metabolites retain significant affinity and activity at CB_1_ receptors (4, 6–8). JWH-073 has been introduced on the illegal market after JWH-018 was recognized as an addictive substance, and therefore, it was banned in 2008 (9). JWH-073 produces effects qualitatively similar to JWH-018 (5), while its potency is roughly 50% less. This difference is related to the binding sites of CB_1_ receptors, which is the chemical structure of JWH-018 is more efficient than JWH-073 (10). JWH-073 has a prolonged effect compared to JWH-018, because of its extended half-life and less receptor internalization (11). JWH-073 pharmacological features, together with its milder effect let users pensive these compounds as safer and more attractive. Thus, it leads to less careful and higher abuse. JWH-073 is often used as an ingredient of herbal blend preparations commonly named “Spice”, alone or mixed with other SCBs, like JWH-018 or JWH-250 (5, 12). Beyond these mixtures induce “desired” psychoactive effects, other significant psychiatric and physical adverse effects: agitation/anxiety, restlessness, acute psychosis, hallucinations, photophobia, hyperreflexia, unconsciousness, panic, confusion, drowsiness, and alterations in cognitive abilities (13–19). These can easily involve psychological issues such as recurrent psychotic episodes in vulnerable subjects (14, 20). Furthermore, more intense effects of these mixtures have manifested as serious sympathomimetic-like symptoms such as psychomotor agitation, diaphoresis, palpitations, tachycardia, tachyarrhythmia, hyperreflexia, and generalized convulsions (14, 15, 21). Preclinical studies report that JWH-073 reproduces the typical “tetrad” (hypothermia, analgesia, hypolocomotion, akinesia) effects previously ascertained for Δ^9^-THC (3, 4, 20. 21), as well as neurological effects, are worse than this latter (22). Moreover, JWH-073 facilitates dopamine release in the shell of the nucleus Accumbens and impairs sensorimotor responses in mice (5). Moreover, it has been shown to affect the drug discrimination paradigm and place preference, in rodents (23–25) and monkeys (26). Along with numerous behavioral effects, SCBs induce EEG hyperexcitability when administered at high concentrations (12). These kinds of alterations apparently include also some typical markers of epilepsy, both *in vitro* (22) and *in vivo* (27). Moreover, SCBs are known to be deleterious to memory functions, affecting neuronal memory mechanisms (28, 29). The introduction into the illegal market of new and powerful SCBs creates the need to study more and more carefully SCBs toxic effects (30–32), and in particular those effects related to functional changes in the central nervous system activity, which can represent the basis of acute or long-term neurological and psychiatric alterations. Indeed, together with a new screening method useful to identify SCBs (33), also preclinical studies need to keep up with the continued expansion of the illegal market. The present study is aimed at investigating the *in vivo* pharmacological activity of acute JWH-073 administration in male CD-1 mice at dosage (1 mg/kg i.p.). The dose chosen is underactive or ineffective on physiological and neurobehavioural parameters, as previously demonstrated in rodents (5, 24). Therefore, we investigate the effect of JWH-073 on sensorimotor (visual, acoustic, and tactile), motor (spontaneous motor activity and catalepsy), and memory functions (novel object recognition; NOR) in mice coupling behavioral and electroencephalogram (EEG) data. To clarify the effects of SCBs on brain electrical activity we recorded EEG from areas potentially involved in epilepsy and sensorimotor impairment, like the hippocampus and somatosensory cortex, analyzing quantitatively EEG at the sub-toxic doses of JWH-073. To investigate if JWH-073 at the tested dose may affect the hippocampal-based synaptic plasticity mechanism such as Long Term synaptic Potentiation (LTP), we used an electrophysiology test on the hippocampus *in vitro* (29, 34, 35).

## Materials and methods

### Animals

Eighty-seven-male ICR (CD-1^®^) mice, 5–7 months old, weighing 35-30 gr (Centralized Preclinical Research Laboratory, University of Ferrara, Italy), were group-housed (five mice per cage; floor area/animal: 80 cm^2^; minimum enclosure height: 12 cm) on a 12:12-h light-dark cycle (light on at 6:30 AM), the temperature of 20–22°C, the humidity of 45–55% and were provided *ad libitum* access to food (Diet 4RF25 GLP; Mucedola, Settimo Milanese, Milan, Italy) and water. Experimental protocols were performed in accordance with the European Communities Council Directive of September 2010 (2010/63/EU) and were approved by the Italian Ministry of Health (license n. 335/2016-PR and license n. 956/2020-PR) and by the Animal Welfare Body of the University of Ferrara. According to the ARRIVE guidelines, adequate measures were taken to reduce the number of employed animals and their pain and discomfort.

### Drug preparation and dose selection

JWH-073 was purchased from LGC Standards (LGC Standards S.r.L., Sesto San Giovanni, Milan, Italy) while the CB_1_ receptor antagonist AM-251 was purchased from Tocris (Tocris, Bristol, United Kingdom). Drugs for *in vivo* tests were initially dissolved in absolute ethanol (final concentration = 5%) and Tween 80 (2%) and brought to the final volume with saline (0.9% NaCl). The solution made with ethanol, Tween 80, and saline was also used as a vehicle. AM-251 (1 mg/kg) was administered 20 min before the JWH-073 injection. Drugs were administered intraperitoneally (i.p.), at a volume of 4 μl/gr. For *in vitro* electrophysiology the substances were dissolved in absolute ethanol (maximum concentration = 0.1% v/v). The JWH-073 dosage of 1 mg/kg was chosen based on previous studies [(5); see Supplementary materials].

### Behavioral studies

#### Evaluation of sensorimotor and motor responses

The effects of 1 mg/kg JWH-073 were investigated using a consolidated battery of behavioral tests routinely used in our laboratory for preclinical investigation of NPSs in rodents (5, 36–39), in a consecutive manner, according to specific time scheme (for technical details, see Supplementary material).

**Figure 1:**
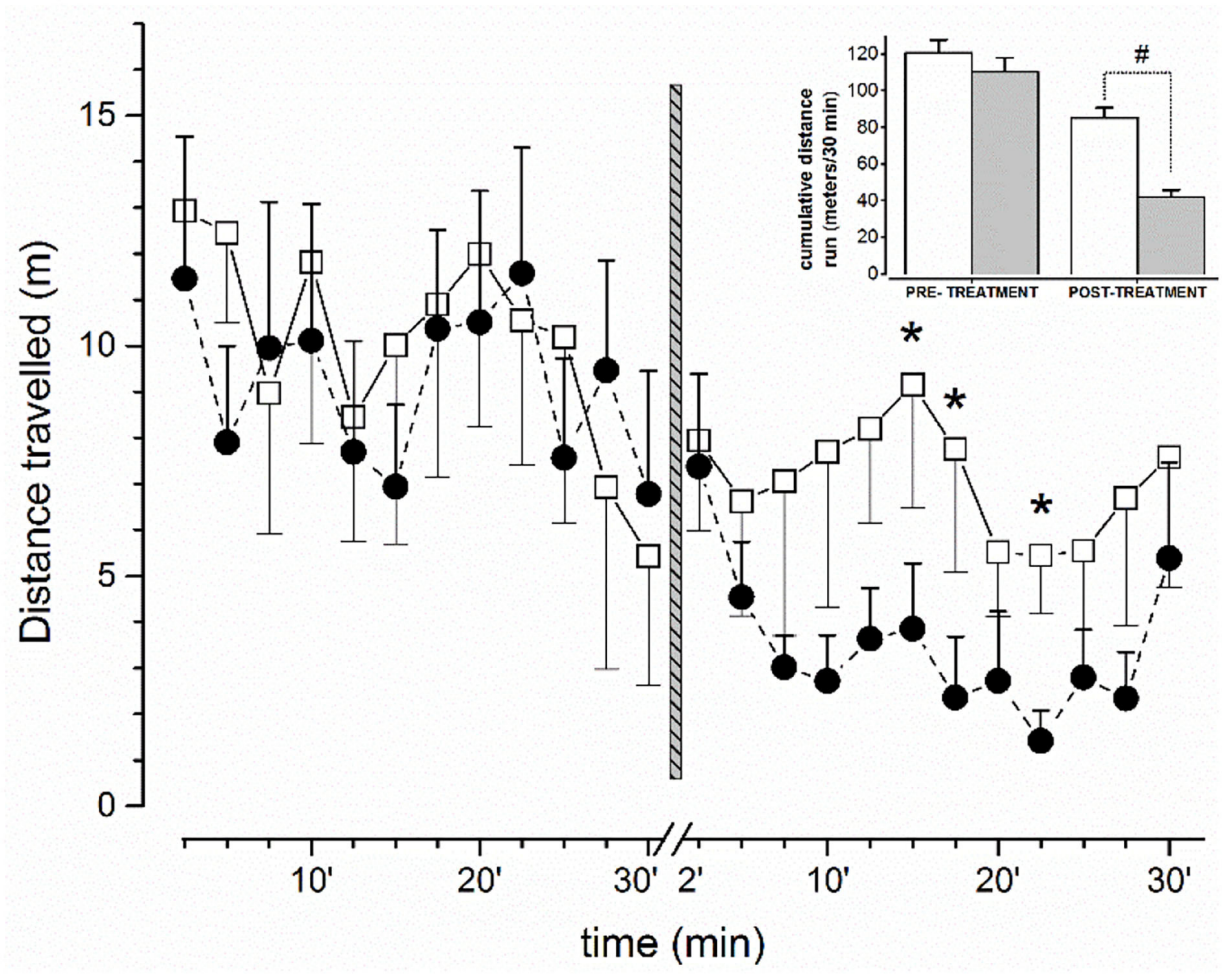
Effect on free locomotor activity of mice before and after administration of vehicle or JWH-073 1 mg/kg. Data compare the average (meter/150 s) distance run before/after vehicle (□, *n* = 5) or JWH-073 (•, *n* = 9) (2.5 min/point). Treatment administration instant corresponds to vertical bar (time 0, middle figure). First 2 min after administration were not recorded. Distance run decreases approximately constantly before treatment and diverges soon after. Inset: Histograms representing cumulative distance run before and after substance administration (White = vehicle; gray = JWH-073). Statistical analysis was performed with t-Student test for unpaired data. *p < 0.05, #*p* < 0.01, vs. vehicle.

Behavioral tests together with EEG recordings were conducted in the same ambient conditions of housed animals. The behavior of mice was previously recorded with a B/W camera (Ugo Basile, Italy) and analyzed off-line in blind. Moreover, in order to analyze and correlate in more detail EEG with the degree of motor activity (potential sudden and temporary motor blocks), the horizontal locomotion of the mouse was video recorded during EEG for off-line analysis (Figure 1). Locomotor parameters were analyzed 30 min before (basal values) and after treatment.

#### Novel object recognition test

In order to evaluate the effect of JWH-073 on the memory functions of the mouse, the Novel Object Recognition (NOR) test was carried out at two-time points (2 and 24 h after the administration) based on the alterations induced by the tested compound on sensorimotor and motor responses (Table 1). As previously reported (29), the test was conducted in three phases: habituation, familiarization, and choice (for technical details of the methods used, see Supplementary material).

**Table 1:** Effects of JWH-073 (1 mg/kg i.p.) or vehicle on spontaneous motor activity, sensorimotor (visual, acoustic and tactile) responses and object memory recognition (NOR) in mice.

**Test**	**treatment**	**Control (−10 min)**	**10 min**	**25 min**	**105 min**	**120 min**	**24 h**
Spontaneous motor activity (s)	Vehicle	165.5 ± 5.5	152.5 ± 6.5	55.2 ± 3.0	29.3 ± 5.1	15.3 ± 5.1	135.6 ± 4.5
	JWH-073	168.7 ± 6.2	**87.3** ±4.6**	**28.5** ±3.2*	25.3 ± 5.6	13.6 ± 4.2	145.5 ± 7.5
Visual object (arbitrary units)	Vehicle	9 ± 0	9 ± 0	9 ± 0	8.5 ± 0.94	8.8 ± 0.74	9 ± 0
	JWH-073	9 ± 0	**5.8** ±0.15**	**5.6** ±0.25**	**6.5** ±0.32*	8.5 ± 0.76	9 ± 0
Acoustic (arbitrary units)	Vehicle	12 ± 0	12 ± 0	11.56 ± 0.3	12 ± 0	11.96 ± 0.2	12 ± 0
	JWH-073	12 ± 0	11.63 ± 0.24	11.55 ± 0.54	11.32 ± 0.31	11.72 ± 0.7	12 ± 0
Overall tactile (arbitrary units)	Vehicle	12 ± 0	12 ± 0	12 ± 0	11.54 ± 0.32	12 ± 0	12 ± 0
	JWH-073	12 ± 0	11.25 ± 0.64	11.47 ± 0.84	11.12 ± 0.21	12 ± 0	12 ± 0
Time on bar (s)	Vehicle	0 ± 0	0 ± 0	0 ± 0	0 ± 0	0 ± 0	0 ± 0
	JWH-073	0 ± 0	0 ± 0	0 ± 0	0 ± 0	0 ± 0	0 ± 0
NORRecognition Index	Vehicle	*nd*	*nd*	*nd*	*nd*	0.29 ± 0.023	0.30 ± 0.024
	JWH-073	*nd*	*nd*	*nd*	*nd*	**0.16** ±0.025**	**0.20** ±0.021*
NORTOE (s)	Vehicle	*nd*	*nd*	*nd*	*nd*	45.5 ± 2.4	38.1 ± 3.2
	JWH-073	*nd*	*nd*	*nd*	*nd*	42.0 ± 4.2	36.0 ± 2.3

Data are expressed as absolute values in seconds (spontaneous motor activity, time on bar and time of object exploration; TOE), arbitrary units (visual, acoustic and tactile responses) and recognition index (novel object recognition test, NOR). Data represent the mean ± SEM of 5 different animals for each treatment. Statistical analysis was performed with two-way ANOVA followed by Bonferroni's *post-hoc* test for multiple comparisons, while the NOR test was performed with *t*-Student test for unpaired data. *p < 0.05, **p < 0.01, vs. vehicle.

Bold values in Table 1 referred to statistically significant variations among all the results.

### Electrophysiological studies *in vitro* in hippocampal slices

#### Tissue preparation

The hippocampal transverse slice model was used to evaluate the acute effects of JWH-073 on synaptic excitatory transmission and plasticity. Tissue preparation protocols are described in detail by Barbieri et al. (29). In brief, after mouse decapitation, the brain was quickly extracted, and the hippocampus was isolated and placed in ice-cold artificial cerebrospinal fluid (aCSF), of the following composition (mM): NaCl, 126; KCl, 2; KH_2_PO_4_, 1.25; NaHCO_3_, 26; MgSO_4_, 2.0; CaCl_2_, 2.5; d-glucose 10. The aCSF was saturated with 95% O_2_/5% CO_2_. Transverse hippocampal slices (425 μm thick) were cut with a tissue chopper, then placed for almost 90 min in a Haas-style incubation chamber for recovery until recording.

#### Electrophysiological recording

A single slice was placed into a recording chamber (3 ml total volume) and continuously superfused (3.0 ml/min) with warmed (33°C) aCSF. Details of recording procedures are described in 27. In brief, once obtained a stable synaptic response from stimulation\recording from CA1 Shaffer collaterals area for at least 20 min, a stimulus/response curve (SRC) was generated as previously described (29) to extrapolate the stimulation intensity (the one achieving the 40% of maximal EPSP) constantly adopted throughout the experiment. Substances (JWH-073, AM251, or vehicle) were added to the reservoir, and the bath was applied *via* the perfusion line. At steady state effect on fEPSP of JWH-073 1 μM, the 40% stimulation amplitude was recalculated, repeating the SRC and adopted for the following LTP procedure.

To evaluate modifications of synaptic plasticity we induced LTP following methods detailed in previously studies (5, 40). At the end of the experimental protocols, pathways were simultaneously activated with TB10 stimulation to evoke the maximally achievable potentiation as a control for slice viability. Amplitude values were used as a reference for the maximal potentiation achievable. The same data was also used to calculate TB5 stimulation-induced LTP as a fraction of maximally inducible potentiation in each slice to equalize differences in LTP between preparations.

### Electrophysiological studies *in vivo*

#### Surgery and electrode implantation procedures

Under initial i.p. anesthesia (ketamine/xylazine, 75/8.25 mg/kg), mice were secured to a digital stereotaxic device (Stoelting, Wood Dale, IL, USA). The mouse skull was shaved, rubbed with the anesthetic cream, and skin incised exposing beneath the bone. Was then drilled a 0.9 mm Ø hole to implant an electrode headset at a stereotaxic position relative to bregma. Electrodes were alternatively positioned in two areas: (1) left frontoparietal cortex - somatosensory area (AP −0.8 mm; ML −1.4 mm; DV −0.5 mm) and (2) left ventral hippocampus (AP −2.0 mm; ML −1.4 mm; DV −1.5 mm). Two further holes were drilled on the same side and loaded just upon the brain with stainless-steel screws used to stabilize the headset to the bone. Finally, dental acrylic was overlaid, embedding screws and covering the inserted headset. For headset building and installation details see the supp documents. Post-surgery pain discomfort and infection were prevented i.p. injecting mice with Tramadol (50 mg/kg) and Imipenem (25 mg/kg, i.p.), for 2 days. On the 7th day of recovery, the headset of the mouse was connected to a swivel system with a 3 wires-cable (Plastics One), and electrode resistance was measured, discarding the implant with electrical resistance, ≤ 1 MΩ. For 5 days before drug/vehicle administration, mice were daily handled, transferred to the recording arena, and connected to a recording device for 1 h to archive EEG later used as control. The signal was delivered to a preamplifier/data acquisition system (EEG100C, MP150, Biopac), allowing the recording of EEG and video synchronously on a PC using AcqKnowledge 4.5 (Biopac) software. The EEG signal was digitized (2 kHz), amplified (from 5 to 50 k fold), and filtered (5 kHz lp, 0.05 Hz hp). EEG baseline was considered stable when relative Fast Fourier Transformation (FFT; 10 s window). showed variations within 10% for a period of almost 10 min. EEG and video were then recorded for 30 min soon after substance injection, then after 2 and 24 h. Drug treatment consisted of intraperitoneal injection of JWH-073 (1 mg/kg; hippocampus *n* = 6; cortex *n* = 5) or vehicle (120 μl at 1 mg/kg, *n* = 5) as sham treatment. Once experimental procedures were terminated, anatomical studies were conducted on all animals to verify the correctness of the implant position.

### Data and statistical analysis

#### Behavioral studies

Data are expressed as absolute values in seconds (spontaneous motor activity, time on bar, and TOE time), arbitrary units (visual object, acoustic and overall tactile responses), and RI in the NOR test. All data are shown as the mean ± SEM of 5 different animals (*n* = 5) for each treatment. Statistical analysis of the effects in behavioral tests over time was performed by two-way ANOVA followed by Bonferroni's *post-hoc* test for multiple comparisons. Analysis of the effect induced by treatment in the NOR test was performed with the unpaired *t*-Student test. The significant level was set at *p* < 0.05. Statistical analysis was performed using the program Prism software (GraphPad Prism, USA).

#### *In vitro* data analysis

Data from *in vitro* recordings has been analyzed as previously described (5). In brief, fEPSP slope was measured to calculate drug effects on synaptic excitatory transmission and TB5 stimulation-induced synaptic potentiation. Steady-state values of net potentiation produced by TB5 stimulation were obtained by averaging the values of the 11 consecutive responses recorded over the 5 min period between 40 and 45 min after TB5 stimulation. The maximally achievable potentiation was calculated by averaging the values of 5 responses over the 2 min period between 13 and 15 min after TB10 stimulation. The unpaired *t*-Student test was used to compare the vehicle-treated with untreated control groups and *p* < 0.05 was considered statistically significant.

#### *In vivo* EEG data analysis

EEG data analysis was performed off-line with AcqKnowledge 4.5 software (BIOPAC, USA). EEG showing components like interictal and/or spikes, spike and wave complexes, or poly-spikes, were defined as “events” when amplitude was at least 2-fold the baseline defined as 20 s trace immediately before and long 2 s or more (41). Spectrograms and FFT (used to quantify EEG power spectrum) were calculated with IgorPro 8.0 software. For spectrograms has been used the Time-Frequency toolbox on data previously resampled to 500 Hz and digitally HP filtered at 0.05 Hz. Quantitative EEG was calculated on a 5 s window. Power for each characteristic EEG band [delta (δ) = 0.3–4 Hz; theta (θ)= 4–8 Hz; alpha (α)= 8-13 Hz; beta (β) = 13-30 Hz; gamma (γ) = 30-48 Hz)] was computed in 10 s bins from FFT for each animal and averaged into 1 min bins when necessary. When calculated, spectral edge frequency (SEF) was 95%.

Two-way ANOVA with the Dunnet test was used to compare the control EEG amplitude of groups before treatment with values after treatment. *P* < 0.05 was considered statistically significant.

## Results

### Behavioral studies

#### Evaluation of sensorimotor and motor responses

Sensorimotor (visual, acoustic, and overall tactile) and motor (time on bar, spontaneous activity) results are reported in Table 1. Sensorimotor parameters were unmodified in vehicle-treated mice and the effect was similar to that observed in naïve untreated animals (data not shown). Systemic administration of JWH-073 (1 mg/kg i.p.) reduced the visual object response in mice of ~36% (at 10 min), ~38% (at 25 min), and ~24% (at 105 min) in comparison with vehicle-treated mice [effect of treatment (F_1,48_ = 36.07, *p* < 0.0001), time (F_5,48_ = 7.436, *p* < 0.0001) and time × treatment interaction (F_5,48_ = 6.933, *p* < 0.0001)]. JWH-073 at 1 mg/kg did not affect at any time point the acoustic and tactile sensorimotor responses in mice. The vehicle did not change the bar test response and spontaneous motor activity in mice, and the effects were similar to that observed in naïve untreated animals (data not shown). Systemic administration of JWH-073 (1 mg/kg i.p.) did not cause catalepsy/akinesia in the bar test for up to 24 h. Conversely, JWH-073 reduced spontaneous motor activity in mice at about ~43% (at 10 min) and ~48% (at 25 min) respect vehicle-treated mice [effect of treatment (F_1,48_ = 21.97, *p* < 0.0001), time (F_5,48_ = 309.9, *p* < 0.0001), and time × treatment interaction (F_5,48_ = 14.28, *p* < 0.0001)].

#### Novel object recognition test

To investigate whether the novel synthetic cannabinoid agonist JWH-073 affects memory retention in mice, the NOR test was performed, comparing results with vehicle (Table 1). During the familiarization phase, no difference was seen in the time spent by vehicle untreated-mice to investigate the two objects, and there were no significant differences in the NOR test, similarly to naïve untreated mice (data not shown). In contrast, treatment with the synthetic cannabinoid-induced a significant impairment of recognition memory. In fact, significant changes in RI values were observed at 2 h (*t* = 3.729, df = 8, *p* = 0.0058) and at 24 h (*t* = 2.820, df = 8, *p* = 0.0225) from the adm inistration of JWH- 073. Clearly, no differences in TOE time between the untreated control animals and vehicle-treated mice (data not shown) were observed. The TOE time in the choice phase was not impaired both at 2 and 24 h after the JWH-073 injection.

#### Spontaneous horizontal locomotor activity during EEG recordings

The amount of spontaneous horizontal locomotor activity of mice before (30 min) and after (30 min) treatment with vehicle or JWH-073 is shown in Figure 1. After 5 min upon JWH-073 administration, the locomotion is dramatically reduced, resulting in a splitting of the two curves (vehicle vs. JWH-073) immediately after treatment. After ~23 min, the curve relative to JWH-073 treated mice displays a return to values relative to vehicle-treated animals' locomotion. The comparison of the cumulative distance traveled by vehicle- and JWH-073-treated groups are summarized in the inset emphasizing the significant difference in the cannabinoid effect to inhibit locomotion. It is worth noting that mice treated with JWH-073 were free of behavioral patterns of pathological significance following Racine stages (42).

### *In vitro* electrophysiological studies

#### Effects of JWH-073 on synaptic transmission in CA1 hippocampal area

**Figure 2:**
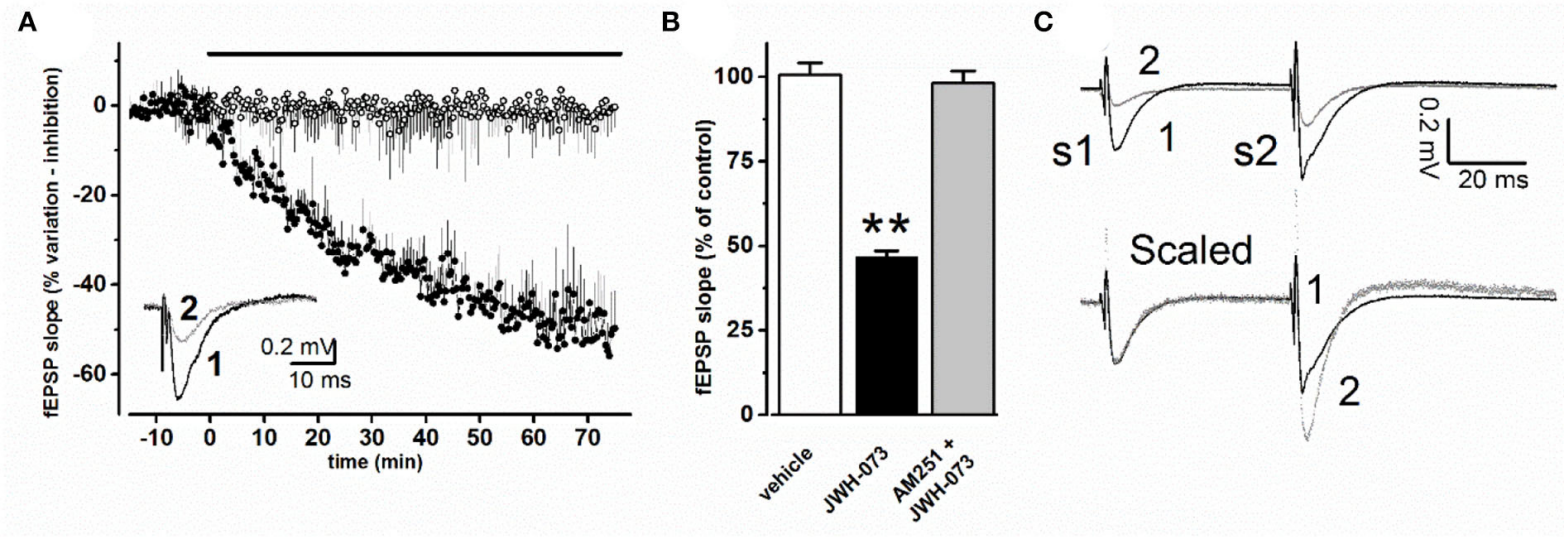
**(A)** Time course effect of JWH-073 1 μM on fEPSP of CA1 area of mouse hippocampal slice. Each point corresponds to the average value of fEPSP slope recorded at the same corresponding time, as % value ± s.e, with respect to average value recorded before drug or vehicle application (black bar above data). (○) = vehicle (*n* = 8); (•) = JWH-073 1 μM (*n* = 13). Inset: representative fEPSP recorded during the same experiment: (1, **black line**) = control, 5 min before drug application; (2, **dotted line**) = 60 min perfusion with 1 μM JWH-073. **(B)** Average fEPSP peak values at steady state as % ± s.e. changes vs. control as average of fEPSP 10 min before drug. When AM-251 was applied, JWH-073 1 μM was the test drug. *n* = (vehicle = 8, white bar; JWH-073 1 μM = 13, black bar; AM251 + JWH-073 = 2, gray bar). Statistical analysis: *t*-Student test for unpaired data. **p < 0.01, vs. vehicle. **(C)** Superimposed representative traces from the same experiment, recorded applying the PPF (paired pulse facilitation) paradigm. Upper traces refer to: (1) control recording (vehicle), black trace; (2) 60 min of JWH-073 1 μM, gray trace. Lower traces (Scaled) are the same of above but after normalization at first fEPSP, allowing comparison of PPF effect on the second stimulus response.

Changes in the average slope amplitude of evoked CA1 synaptic population response are represented in Figure 2A. JWH-073 in all tests analyzed, induced a depressive effect on fEPSP, declining from 2 to 5 min after first contact with JWH-073 1 μM, with steady state maximal effect after about 60 min. The effect of the vehicle is reported for comparison. Figure 2B are compared steady-state values of fEPSP (vehicle: 100 ± 3.6; JWH-073: 46.7 ± 1.8), with a statistically significant difference (*p* < 0.01). Application of AM-251 (Figure 2B) confirms the selectivity of JWH-073 on CB1 receptors.

#### Effects of JWH-073 on paired-pulse stimulation and fiber volley in CA1 hippocampal area

A paired-pulse stimulation protocol was used to test for pre- or postsynaptic effects of JWH-073 (Figure 2C). At JWH-073 steady-state effect, the ratio between fEPSP slope of conditioning (S1) and test pulse (S2) (Figure 2C, upper, trace 2) was modified with respect to vehicle ratio (Figure 2C, upper, trace 1) accounting for a direct presynaptic effect. The fiber volley amplitude comparing JWH-073 to vehicle effect, showed as it was unmodified (data not shown).

#### Effect of JWH-073 on synaptic plasticity in the CA1 hippocampal area

**Figure 3:**
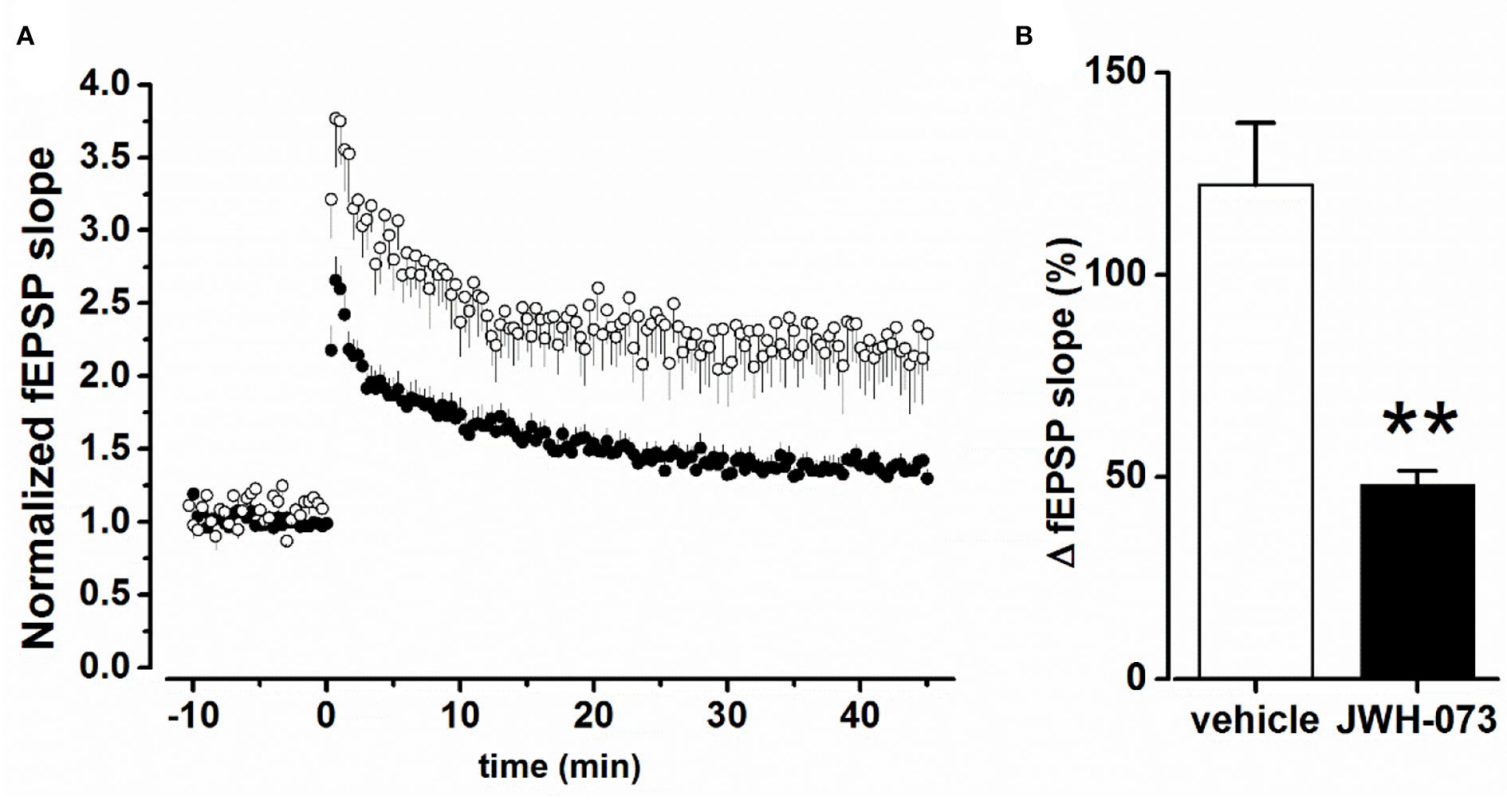
**(A)** Time course of fEPSP after TB5 tetanic stimulation inducing LTP. (○) = control/vehicle values normalized vs. pre LTP average amplitude (*n* = 6); (•) as above at steady state effect of JWH-073 1 μM (*n* = 13). **(B)** Averaged steady peak fEPSP at stable LTP. Data as fEPSP % slope ± s.d., comparing control/vehicle (hollow bar) and after JWH-073 (black bar). **p < 0.01 with *t*- Student test for unpaired data.

Figure 3A shows superimposed normalized average experimental points of LTP test experiments, in the presence of the vehicle or JWH-073. Figure 3B summarizes values at a steady fEPSP slope for the two conditions considered, showing a significant statistical difference (*p* < 0.01). When compared to the vehicle (122.3 ± 15.3% Δ increase vs. fEPSP slope baseline), JWH-073 reduced the development of LTP (48.1 ± 3.5%), impairing the formation of any consistent stable synaptic potentiation. In slices treated with JWH-073, the TB10 saturating test (see Methods) was unable to induce any further potentiation, confirming the strong LTP-inhibition from JWH-073 treatment (data not shown).

### *In vivo* electrophysiological studies

#### Effect of JWH-073 on *in vivo* EEG in cortical area

**Figure 4:**
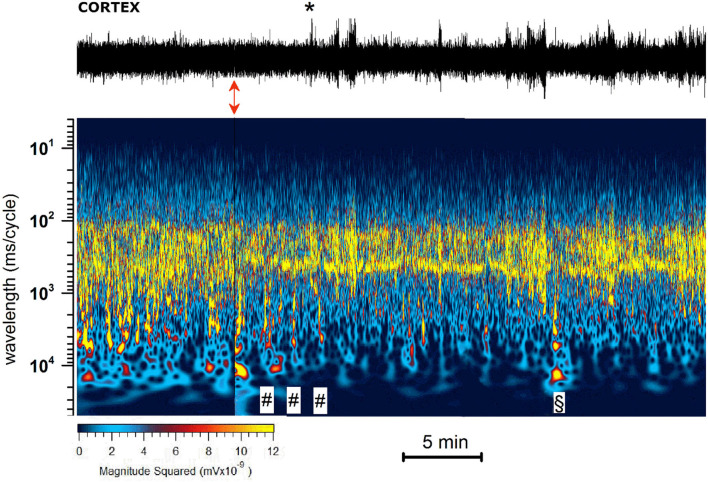
Representative 40 min recording of cortical EEG during JWH-073 administration and relative spectrogram. **(Upper)** Raw EEG with JWH-073 administration (red double headed arrow). Asterisk points to the first high amplitude event. **(Bottom)** Corresponding spectrogram correlating time (X) vs. wavelength (Y) and power (color scale). Wavelength ranges from 0.05 to 150 Hz (Y axis, bottom to top). Color power scale (below X scale), with power magnitude increasing from left to right (smallest to highest values = darker to lighter colors). “#” symbols (bottom of the spectrogram), correspond to short-high energy episodes with frequency range 0.7–7 Hz (not visible in the corresponding above EEG). “§” symbol (20 min) points to an event with frequency range ~ 2 to 30 Hz followed by a short depression then by high energy/low frequency (0.05–7 Hz) burst events. Similar sequelae were present in all experiments.

**Figure 5:**
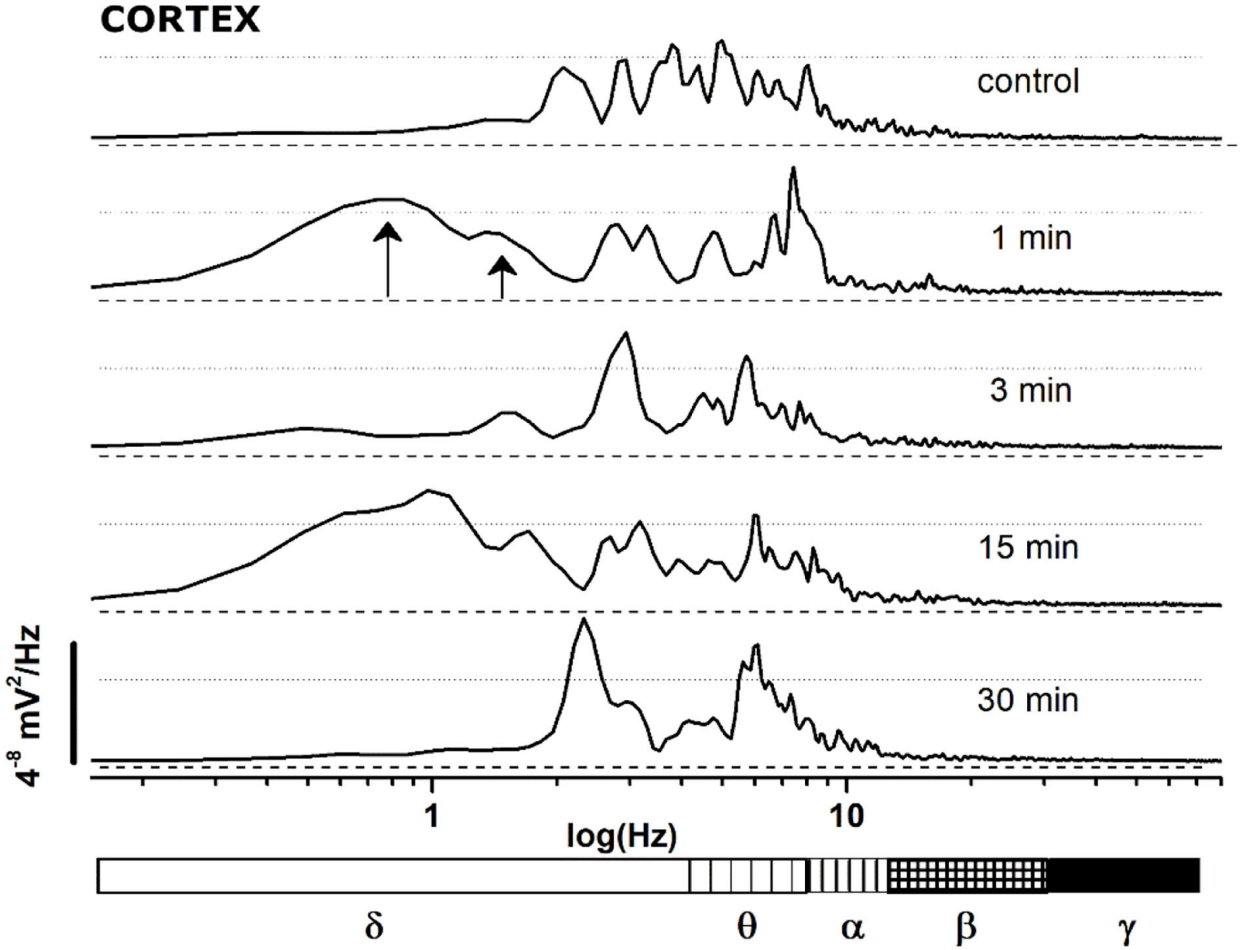
FFT spectrum of a 30 s window, sampled at definite time points (control and minutes 1, 3, 15, 30 after JWH-073 administration) from EEG recording of Figure 4. Textured bars under the X axis correspond to the different EEG bands indicated below.

**Figure 6:**
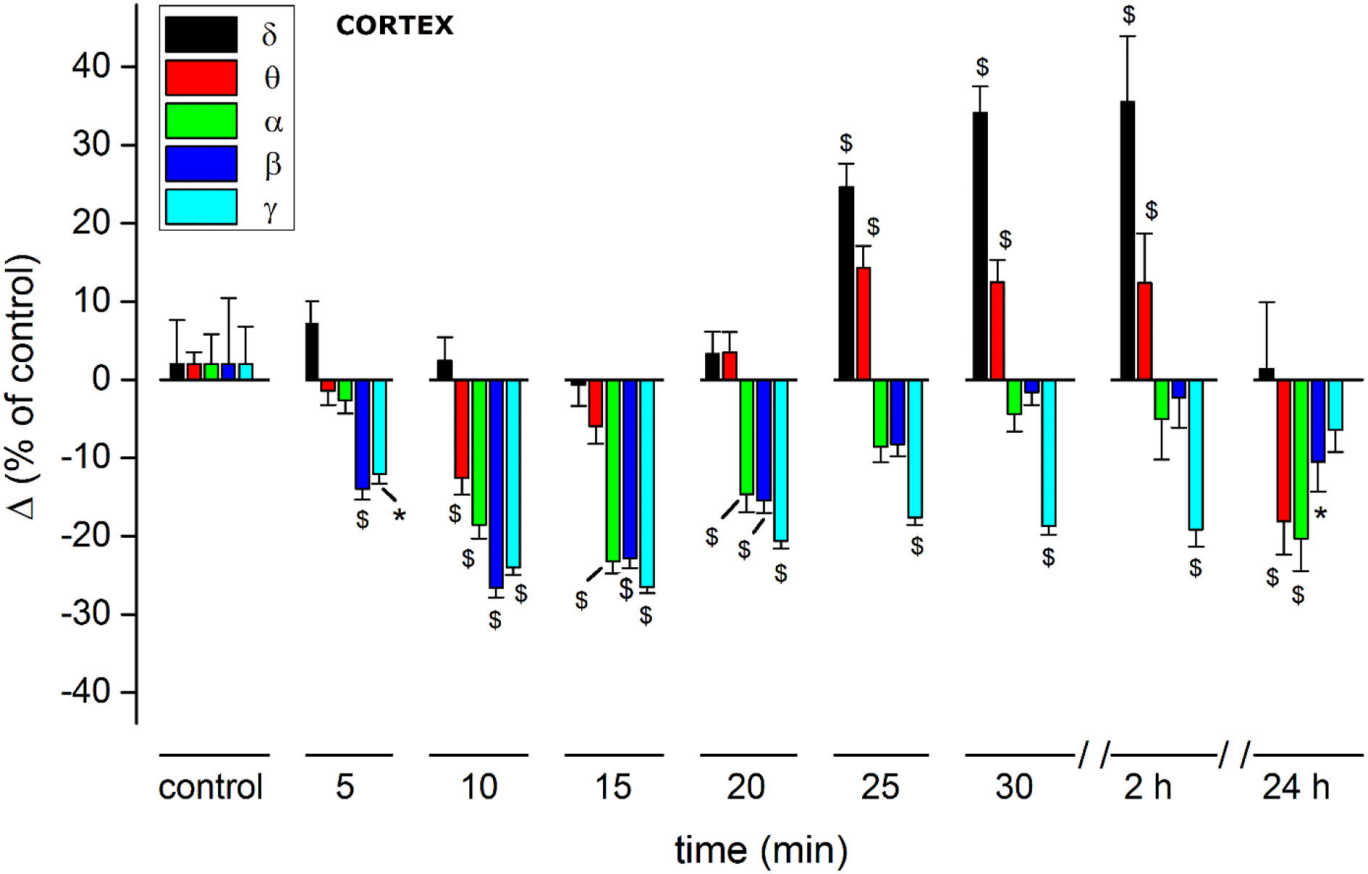
Histogram summarizing average relative % changes of δ, θ, α, β, γ, amplitude, measured as FFT area relative to a 5-min window and recorded in somatosensory cortical area. Histograms at control are exclusively functional to mate EEG color bands with corresponding error bars. Values correspond to % ± s.e. Original data are reported in Supplementary materials and Table 2. Two-way ANOVA with Dunnet test used to compare the control EEG amplitude of groups before treatment with values after treatment. *p < 0.05; ^$^*p* < 0.01.

As illustrated by the representative experiment shown in Figure 4, JWH-073 1 mg/kg administration evoked a series of complex and rapid modifications in cortical EEG and precisely identified by the spectrogram.

(A) JWH-073 modifies clearly EEG (Figure 4) after a few minutes (in this case, 5 min) from administration, with short, high amplitude episodes (asterisk marked) followed by similar throughout the whole recording. EEG spectrogram (Figure 4) unveils how after a few minutes from the drug an evident power reduction of α, β, and γ frequencies bands. EEG power trends to recover with time, with increased incidence of high amplitude events. Interestingly, soon after the drug injection, it's usual to observe some δ bursts, without corresponding EEG evidence.
(B) To characterize the evolution of the power spectrum in Figure 4, we derived the FFT for windows of the 30 s at defined time points (Figure 5): control (recorded before the drug administration); after administration of JWH-073 at 1, 3, 15, and 30 min. Already at point 1 min, the δ band increases macroscopically, splitting out into two sub-components [arrows labeled; Figure 5; (43)]. Subsequently, this slow δ component amplitude oscillates along the recording while the faster remained roughly constant throughout, here and in all the animals tested about a broad power depression of mild entity interspersed with short powerful δ bursts.
(C) For a quantitative EEG analysis, we normalized FFT amplitude measured in a 10 s window for each frequency component, averaging the results in blocks of 5 min (Figure 6; Table 2) and comparing absolute and relative spectrum variations for different animals. Arose clearly three trends: (1) δ and θ amplitudes from 25 min after JWH-073 until 2 h, which showed a significative gradually increasing trend. (2) β and α faced a significant fall between 5 and 20 min, eventually returning to control. (3) γ significantly depressed from 5 min after JWH- 073 effects which persisted almost for 2 h after treatment, extending effects after 24 h. Indeed θ, α, and β were significantly depressed, while other bands completely recovered.

**Table 2:** Quantitative EEG cortex.

**EEG band**	**Control**	**5 min**	**10 min**	**15 min**	**20 min**	**25 min**	**30 min**	**2 h**	**24 h**
Delta	0 ± 5.64	7.17 ± 2.85	2.45 ± 2.99	−0.65 ± 2.69	3.33 ± 2.81	24.63 ± 3.03^$^	34.12 ± 3.42^$^	35.57 ± 8.41^**$**^	1.40 ± 8.50
Theta	9 ± 1.52	−1.40 ± 1.80	−12.55 ± 2.17^**$**^	−5.95 ± 2.27	3.50 ± 2.61	14.28 ± 2.86^**$**^	12.48 ± 2.83^**$**^	12.36 ± 6.32^**$**^	−18.16 ± 4.22^$^
Alpha	0 ± 3.82	−2.61 ± 1.70	−18.60 ± 1.72^$^	−23.25 ± 1.55^$^	−14.64 ± 2.28^$^	−8.60 ± 1.96	−4.35 ± 2.29	−5.03 ± 5.21	−26.29 ± 4.15^$^
Beta	0 ± 8.48	−13.98 ± 1.38^$^	−26.67 ± 1.15^$^	−22.81 ± 1.28^$^	−15.49 ± 1.55^$^	−8.27 ± 1.55	−1.57 ± 1.67	−2.29 ± 3.87	−10.48 ± 3.85*
Gamma	0 ± 4.83	−12.07 ± 1.21*	−24.05 ± 0.88^$^	−26.53 ± 0.75^$^	−20.63 ± 0.94^$^	−17.63 ± 0.93^$^	−18.73 ± 1.12^$^	−19.18 ± 2.18^$^	−6.42 ± 2.85

Values relative to histograms in figure 6 with relative significance. Values correspond to % ± s.e. Two-way ANOVA with Dunnet test used to compare the control EEG amplitude of groups before treatment with values after treatment. *p < 0.05; ^$^*p* < 0.01.

#### Abnormal excitability in cortical EEG after JWH-073

**Figure 7:**
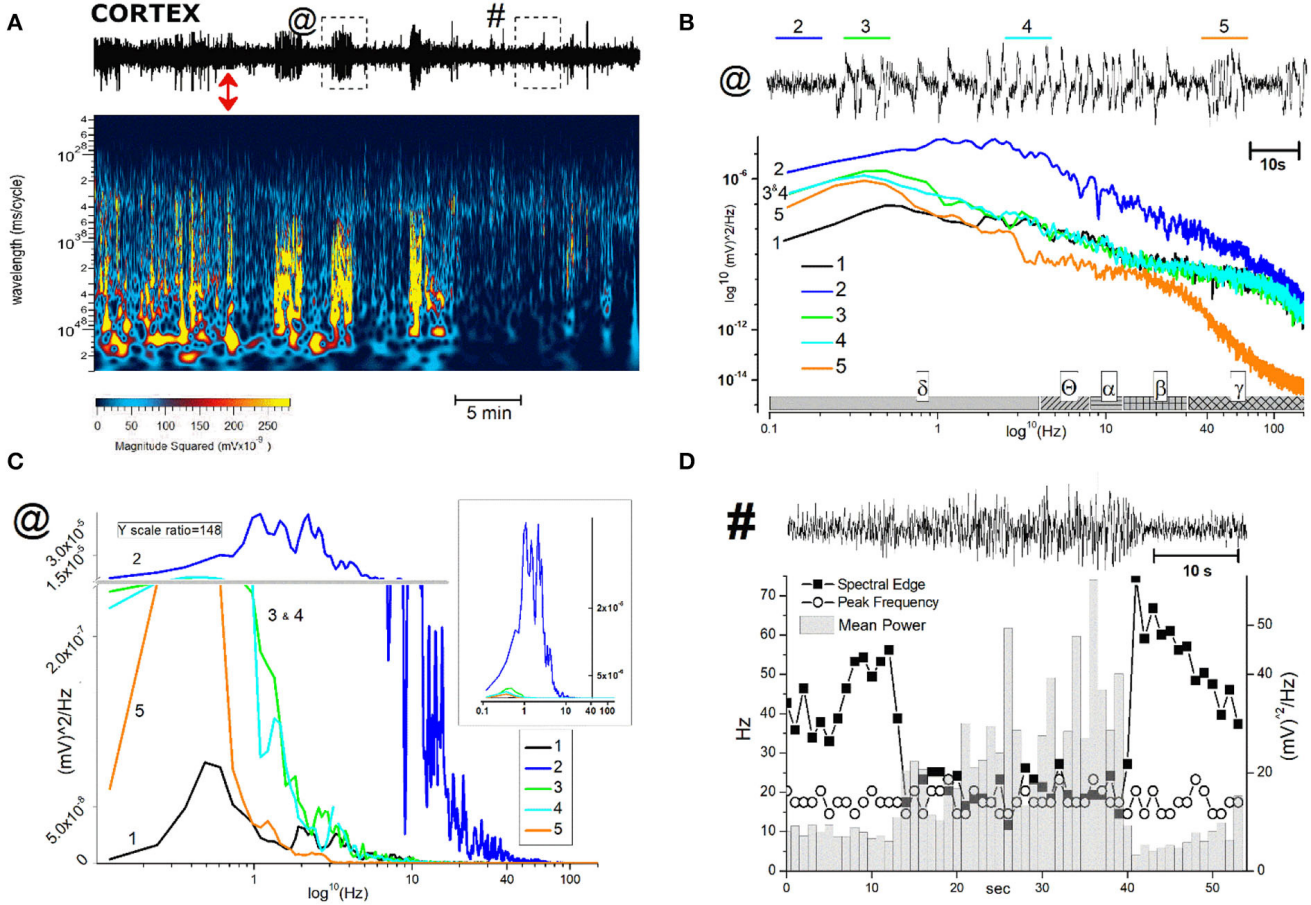
Description and analysis of unusual cortical EEG events after JWH-073 administration. **(A)**, Top: representative 40 min EEG recorded from somatosensory area, before and after JWH-073 administration (marked with a red double headed arrow). Framed areas in EEG, labeled with “@” and “#” and analyzed in detail in panel **(B,C)** (for @) and **(D)** (for #), are EEG traits showing relevant difference respect to control. **(A)** (center): Spectrogram of above EEG. **(B)** EEG area “@” of panel **(A)** is replayed on the top at a faster time scale. Tracts labeled from 2 to 5 (named in text as “complex”), mark altered electrical activity different each other and from control (referred as 1, not shown in EEG). **(B)** (middle): power spectrum analysis of 1–5. The X axis has a scheme bar referring to EEG bands. **(C)** Same plot of **(B)** adopting linear Y scale; Y broken (horizontal gray line), marks different scale ratio (1/148 of the bottom scale) adopted to constrain all plots in a limited ordinate. **(B)** Inset: Same figure but without the Y axis break. **(D)** (top): EEG area “#” of panel **(A)** is replayed on the top at a faster time scale. Left Y axis (Hz) refers to “spectral edge” and “peak frequency”; right Y axis refers to “mean power”.

Different authors agreed on the epileptogenic activity of SCBs (27): coherently with these observations we selected and analyzed two representative events because potentially early markers of epileptic activity (44). To define if EEG spikes labeled with asterisk in Figure 4, could be markers of epileptogenic activity, we analyzed similar events in a representative recording (Figure 7). The EEG trace on the upper part of panel A refers to a recording coping pharmacological protocol of Figure 4. The effect of JWH-073 on EEG is evident, similarly to those described in Figure 4 and exploited by spectrogram. The spectrum shows initial phases of generalized depressed electrical activity (darker areas), alternated with short powerful events (yellow bands). The later part of EEG is characterized by a deeply dampened power spectrum. For the analysis of possible background pro-epileptic electrical components, we selected two representative areas, characterized by evident higher electrical activity on EEG, ideally epileptogenic and marked by “@” (3 min after JWH-073 administration), and “#” (20 min after JWH-073 administration). To analyze the “@” area (Figure 7B), we marked four areas of interest (from 2 to 5) having similar amplitudes, but different intrinsic frequencies. Control is area 1, recorded before JWH-073 and not shown as being outside of figure boundaries. Analysis of these segments marked a common δ increase and segment 5, is characterized by a γ collapse. Real changes could be misread, by using a logarithmic Y-axis. Indeed, panel C replays the same FFT using a linear Y-axis which was broken to include two upper peaks. The inset shows the spectrum of panel B area 2, with control FFT hindering all the other FFT (Figure 7C). To sum up, this pattern of bursts, frequent during the experiment, is mainly characterized by hovering δ episodes coped with short γ depression. The second waveform marked with “#” (Figure 7D), and frequent after JWH-073, is characterized by short, high-frequency bursts and an electrical oscillation of gradually increasing amplitude, ending suddenly later with a short dampened electrical activity. Similar sequelae are characteristic of the pre-epileptic ictal events previously quoted. Spectral analysis (panel D, bottom) described a stable peak frequency with a fall of spectral edge frequency (SEF) (≈ −70%) and a synchronous gradual increase in mean power. During the later EEG attenuation, the power magnitude halved with a sudden peak of SEF. Taken together, differently from seizure-like events, the analysis shown in Figures 7B–D differs from interictal activity, characterized by definite frequency increase with the rise of power magnitude (44).

#### Effect of JWH-073 on *in vivo* EEG in hippocampal area

**Figure 8:**
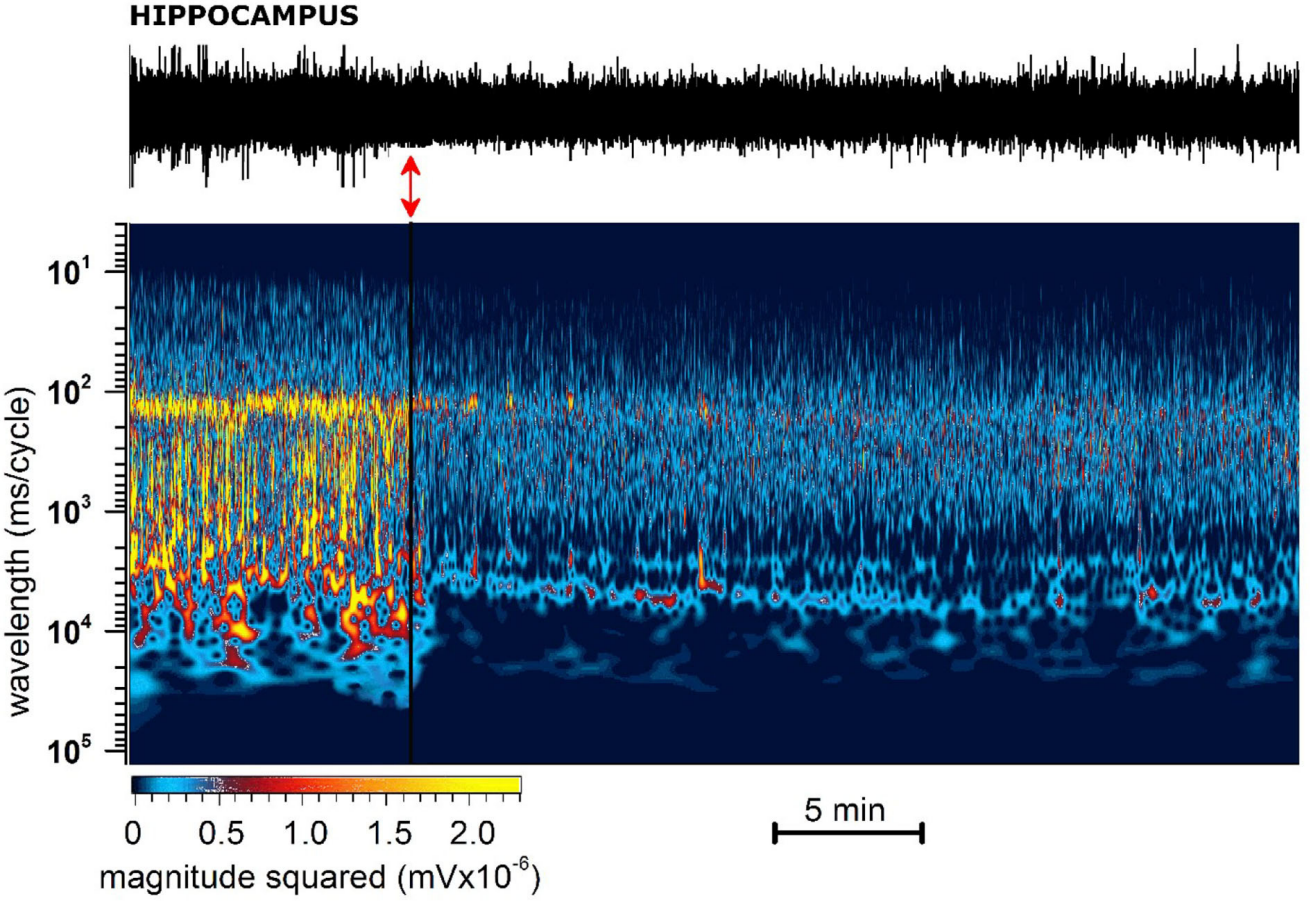
Representative 40 min recording of hippocampal EEG with JWH-073 administration and relative spectrogram. **(Top)** Raw EEG with JWH-073 application (red double headed arrow). **(Bottom)** Corresponding spectrogram correlating time (X) vs. wavelength (Y) and power (color scale). Wavelength ranges from 0.05 to 150 Hz **(bottom to top)**. Color power scale (below X scale), with power magnitude increasing from left to right (smallest to highest values = darker to lighter colors). Soon after JWH administration is visible a radical and fast modification of the EEG spectrogram by global steady fading of colors toward dark and blue.

**Figure 9:**
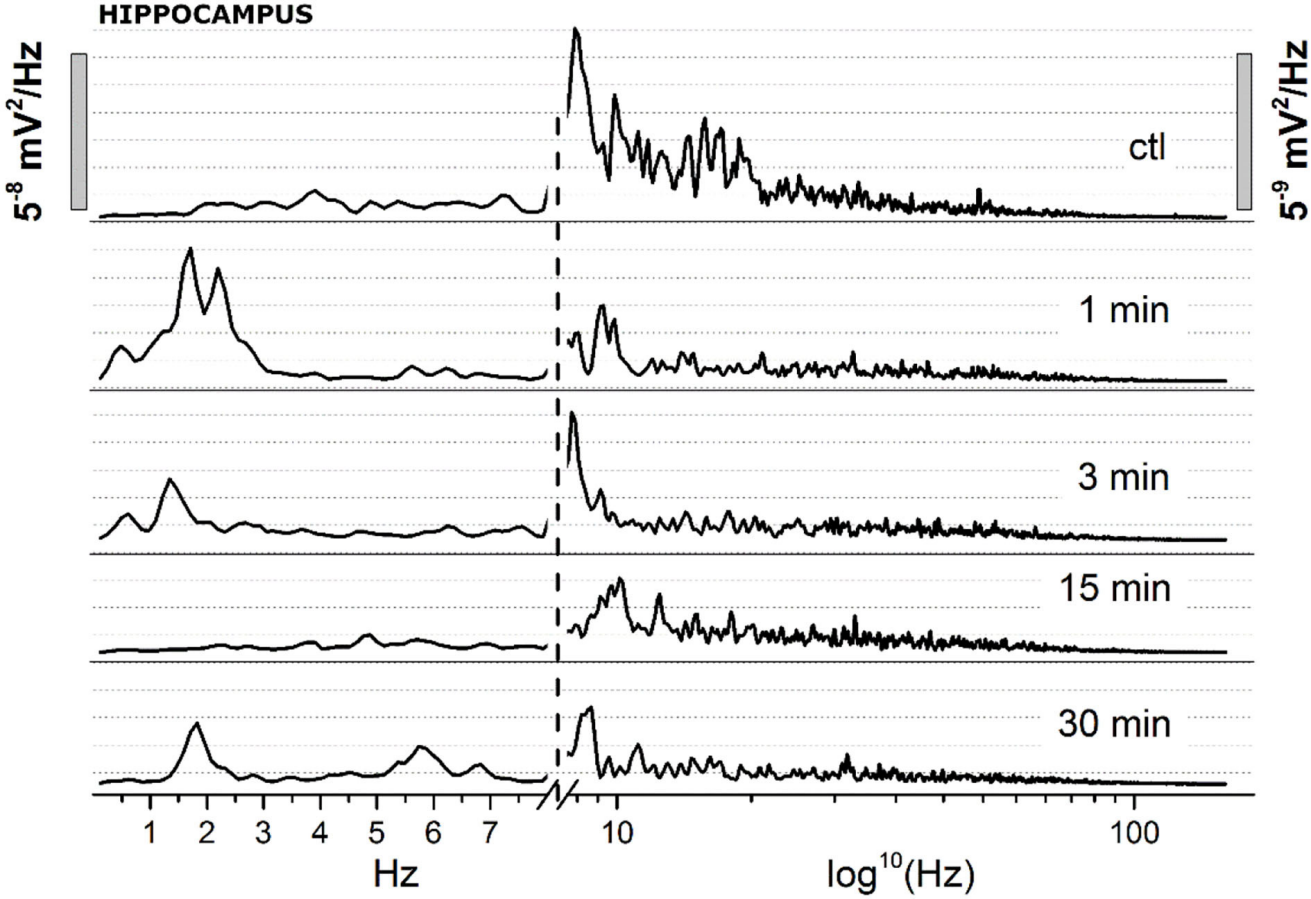
Spectrum analysis (FFT) of 30 s EEG segments of the figure at definite arbitrary time points (1, 3, 15, 30 min). Two different Y scales (right vs. left, 1/10 ratio), has been adopted. The X axis has been broken, with linear values from 0.05 to 8 Hz, and logarithmic from 8.1 to 150 Hz.

**Figure 10:**
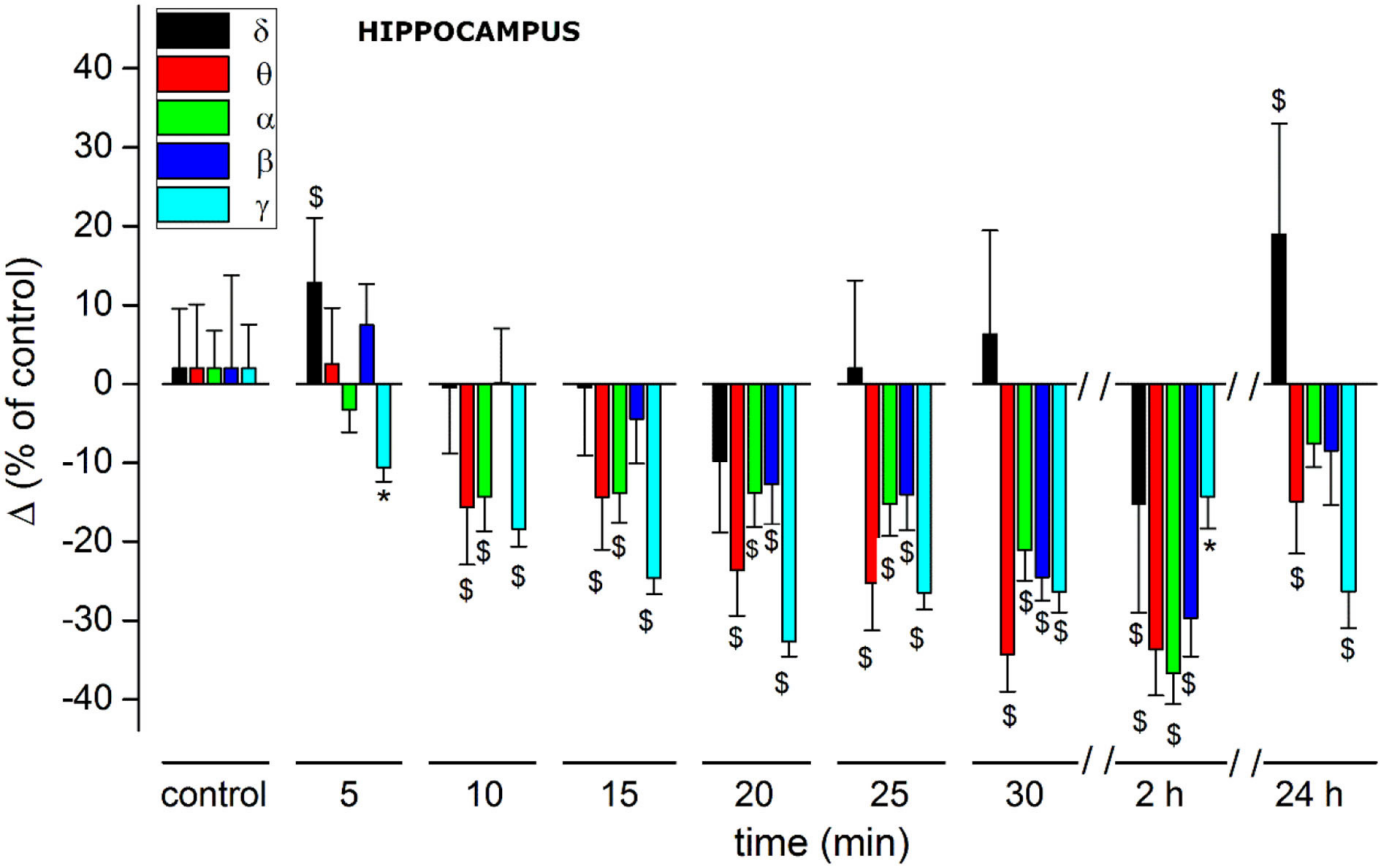
Histogram summarizing average relative % changes of δ, θ, α, β, γ, EEG bands amplitude (measured as FFT area relative to a 5-min window) and recorded in hippocampus. Histograms at control are exclusively functional to mate EEG color bands with corresponding error bars. Values correspond to % ± s.e. Original data are reported in Table 3. Two-way ANOVA with Dunnet test was used to compare the control EEG amplitude of groups before treatment with values after treatment. *p < 0.05; ^$^*p* < 0.01.

(A) Administration of JWH-073 modified hippocampal EEG which differed qualitatively and quantitatively from effects seen for the cortex. Early after JWH-073, EEG amplitude shrank slightly. Spectrum analysis revealed a rapid and persistent fall among all EEG frequency bands (Figure 8).
(B) To focus on how JWH-073 changed EEG bands with time, we sampled the recording at different time points for windows of 30 s whose FFT is represented in Figure 9. The δ band clearly increased at 1 min of JWH-073, decaying partially at 3 an 15 min returned to basal amplitude, when at 30 min δ and θ raised over control. Conversely, all the other bands showed a quickly generalized dampening, mirroring the spectrogram of Figure 8 where, differently from the cortex, any recovery looks absent.
(C) For a cumulative quantitative EEG analysis we adopted identical criteria used for effect of JWH-073 on *in vivo* EEG in hippocampal area, point (C) and Figure 6. Results are reported in Figure 10 and Table 3. The δ band shows an immediate, transitory increase, as seen in Figure 9, returning rapidly to control. All the other bands depress progressively until the recording ends. It is relevant (see Discussion) θ band, which progressively attenuates; γ has a similar trend for the first few minutes as the literature already described. Different from the cortex, in the hippocampus at 2 h after JWH-073 administration, all bands are depressed, recovering 24 h later except for γ and θ. Interestingly, 24 h after JWH-073, δ has an evident amplitude lift compared to other bands determining a net differential effect with δ prevalence over all the others.

**Table 3:** Quantitative EEG hippocampus.

**EEG band**	**Control**	**5 min**	**10 min**	**15 min**	**20 min**	**25 min**	**30 min**	**2 h**	**24 h**
Delta	0 ± 7.54	12.85 ± 8.19*	−0.45 ± 8.40	−0.36 ± 8.73	−9.79 ± 9.05	2.04 ± 11.12	6.34 ± 13.13	−15.22 ± 13.74^$^	19.23 ± 14.02^$^
Theta	0 ± 8.06	2.56 ± 7.07	−15.67 ± 7.30^$^	−14.38 ± 6.63*	−23.62 ± 5.78^$^	−25.30 ± 5.97^$^	−34.25 ± 4.76^$^	33.68 ± 5.84^$^	−14.9 ± 6.62*
Alpha	0 ± 4.74	−3.27 ± 2.86*	−14.34 ± 4.32*	−13.90 ± 3.76*	−13.80 ± 4.38*	−15.27 ± 3.99^$^	−21.08 ± 3.87^$^	−36.66 ± 3.93^$^	−7.60 ± 2.94
Beta	0 ± 11.76	7.505.23	0.17 ± 6.85	−4.43 ± 5.68	−12.76 ± 5.03*	−14.05 ± 4.46*	−24.51 ± 2.93^$^	−29.71 ± 4.86^$^	−8.49 ± 6.86
Gamma	0 ± 5.50	−10.63 ± 1.78	−18.40 ± 2.23^$^	−24.60 ± 2.09^$^	−32.67 ± 1.90^$^	−26.50 ± 2.10^$^	−26.40 ± 2.58^$^	−14.32 ± 4.06*	−26.34 ± 4.62^$^

Values relative to histograms in figure 10 with relative significance. Values correspond to % ± s.e. Two-way ANOVA with Dunnet test used to compare the control EEG amplitude of groups before treatment with values after treatment. *p < 0.05; ^$^*p* < 0.01.

#### Anomalous excitability in hippocampal EEG after JWH-073

**Figure 11:**
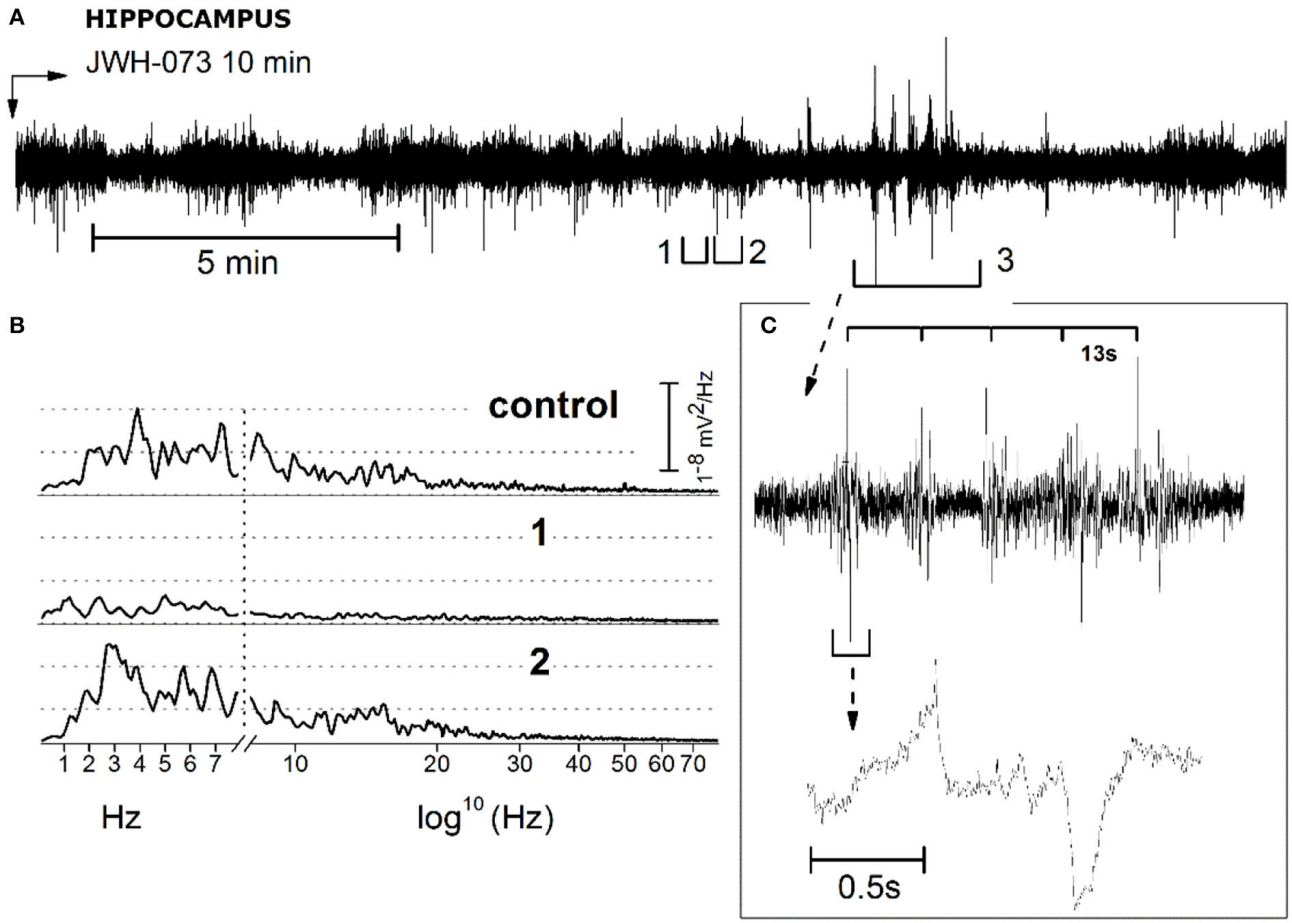
Description and analysis of unusual electrical EEG hippocampal activity resulting from JWH-073 administration. **(A)** Representative EEG recording. Segments tagged as 1 and 2, points to characteristic short traits frequently present only after JWH-073 administration. Panel **(B)** compares the FFT of control (recorded soon before JWH-073), complex 1, complex 2. Similar to Figure 9, X axis has been broken: linear between 0.05 and 8 Hz, logarithmic from 8.01 to 150 Hz. **(C)** Analysis of segment 3. Top: segment “3” at a faster time scale. Time scale bar above the trace identifies a spiking rhythmicity (13 s). Bottom: one of these upper events, shown at a faster speed.

JWH-073 treatment induced in the hippocampus a sporadic series of electrical anomalous events. Figure 11A refers to a recording with some representative events, arbitrarily selected (tagged from 1 to 3) for analysis. Events 1 and 2 are characterized by a definite pattern repetitively present in all recordings. Indeed, 1 shows an alternation of short, low amplitude electrical signals, and 2 has higher amplitude spikes. Event 3 is a group of repetitive spikes, resembling interictal epileptic-like events. Figure 11B represented a comparison of 10 s FFT between control, events 1 and 2. FFT of 1 unveils a consistent generalized depression, while in 2 the δ component is prevalent. Events 1 and 2 alternance retraces a condition already observed in the cortex (Figure 5), where phases of global depression interspersed with others where δ overcomes the amplitude of control. Complex 3, resembles epileptiform interictal activity: high amplitude regular spiking with a “spike and wave” pattern. Figure 11C reproduces this waveform showing a rhythmic activity (13 s phase). Analyzing one complex at a higher speed (Figure 11C, lower part), it differs from fingerprint of the epileptic-ictal spike (45, 46), with a waveform shape alike pre-ictal event, but much slower. Furthermore, spike amplitudes are 2–3 times that of basal EEG too small to be an epileptic focus. As previously verified, JWH-073 has no evident epileptogenic capabilities even at a 30-fold higher dosage, in rats (22), while hippocampal EEG is modified toward hyperexcitability.

## Discussion

This study showed that acute JWH-073 (1 mg/kg; i.p.) administration produces relevant and long-lasting *in vivo* EEG alterations in the somatosensory cortex and hippocampus of mice, which seem to be associated with mild and transitory impairment of visual sensorimotor response and locomotion, and a persistent memory functions compromission. These results, together with *in vitro* in hippocampal slices data, underlined the detrimental activity of JWH-073 upon memory mechanisms and brain electrophysiological activity.

### Behavioral and locomotor effects

JWH-073 (1 mg\kg) transiently inhibited the visual object response, reduced overall motor activity, and deteriorated the long-lasting working memory. This suggests that visual and motor impairments, albeit in a transient way, are the effects firstly appearing from mild dose intake of this SCB, rising the relevance for the correlation between impaired psychomotor activity (e.g., driving) and modest SCBs intoxication (47, 48). In fact, impaired visual responses with depressed memory functions alter the subject's ability to react quickly to dangerous situations which require a rapid sensorimotor response, and the recruitment of previously acquired and stored information. Specifically, JWH-073 depressed working memory in the NOR test (2 and 24 h post-administration), similarly to other SCBs (29, 49). Despite this, TOE time exclusive memory-cognitive deficits induced by JWH-073, alike other SCB (49), showing its effects on processes involved in memory formation and retention rather than the impairment of motor activity. The transient reduction by JWH-073 of the distance traveled parameter supported the previous observation. Indeed, Ossato and colleagues, showed that AM-251 prevented the psycho-cognitive and memory effects of JWH-073, demonstrating its CB1 receptors mediated action (5). Moreover, during the period of reduced mobility, mice still preserved minimal sporadic activity such as random head movements, scratches, or a short walk, which are incoherent with catalepsy. This is in line with previous studies showing the limited influence of JWH-073 on locomotion and catalepsy (23, 50).

### *In vitro* hippocampal electrophysiological effects

On the hippocampal *in vitro* slice model, JWH-073 produced effects like JWH-018 (29) and other SCBs (35) such as irreversible fEPSP inhibition, slow time to onset of a steady state, significant inhibition of early and late LTP components. Pre- and post- synaptic activity of JWH-073 suggest that it is active on different cellular pathways being early LTP regulated by CaMKII activation, while late LTP requires PKA, MAPK, and CREB activation with TrkB-gene expression and functional prions CPEB2-3-mediated translation (51). Thus, it implies *in-vivo* impairment of short- and long-term memory, depicting in perspective a persistent memory deficit following repeated drug use, even at sub-toxic doses (52).

### *In vivo* effects on EEG: General considerations

To date, alterations in EEG induced by Δ_9_-THC has been previously demonstrated in human (53, 54), primates (55), and rodents (56). The EEG alteration after SCBs administration has been investigated only in rodents analyzing EEG exclusively to spot epileptic events, from 30 min after administration and over, and a few of them analyzed EEG spectrum (52, 57–62) and never quantitatively. Since JWH-073 impaired both locomotor activity and visual object response in mice during the first 30 min after treatments, we followed EEG evolution at the same time point, a time context for EEG analysis after CB1R activation previously seldom investigated in detail (57, 62).

Our results demonstrated for the first time that apparently harmless transitory impairment of sensorimotor visual response and locomotor activity, occurs with significant EEG alterations, persisting far beyond the recovery of visual and locomotion impairments. The second novelty which emerges from EEG analysis, is the frequency-band selectivity of these alterations, exploiting a complex spectrum modification discussed below in detail.

#### In vivo effects on EEG: Qualitative and quantitative cortical and hippocampal electroencephalographic analysis

In the sensory cortex, JWH-073 injection produced a severe unbalance between δ, θ, and the other bands within the first 30 min (Figure 6; Table 2). Cortical δ, and θ in a lesser extent, remained unchanged until 20 min after JWH-073 administration, but a gradual highly significant increase follows, persisting for 2 h. Interestingly, the γ band faced a deep and persistent depression, as already described (52, 61). From comparison with other bands' trends, the outcome is a severe unbalance, with a prevalence of δ/θ. Behavioral effects of JWH-073 recovered 2 h later, while the EEG alterations persisted. After 24 h, only δ/γ recovered while θ, α, and β were still depressed. Taken together these results suggest the harmful effect induced by mild doses of JWH-073 on EEG, with alterations persisting much beyond the recovery of pre-drug behavior. JWH-073 depresses all hippocampal EEG bands except for δ, a condition lasting up to 2 h after drug contact, with partial recover 24 h later (Figure 10; Table 3). This is the first quantitative and qualitative electrophysiological analysis in the hippocampal area immediately after an SCB administration. Following data interpretation required correlating dynamically, EEG bands unbalancing, the connection between two brain areas, and behavioral parameters.

#### In vivo effects on EEG: Comparison and hypothesis on correlates of hippocampal/cortical functionality

From the comparison of cortical and hippocampal EEG alterations, stand out three relevant differences\similarities: (1) while in the hippocampus the spread of depression development shows no steady state within the first 30 min of recording, the cortex displays somehow recovery, except for γ. (2) θ drifts in opposite directions: in the hippocampus, it depresses increasingly with time, whereas in the cortex floats from brief mild depression to later highly significant growth. (3) a transitory significant increase of δ power in both areas immediately after the JWH-073 administration. This latter is potential of primary interest even if transitory and less intense as compared to points (1) and (2) and complexity beyond these δ fluctuations is suggested in Figures 5, 7: as frequently seen among all tests, EEG power amplitude alternates macroscopically in time, reflecting a pulsatile irregular alternate of hyper-excitability and depression. Figure 5 overall underlines this fluctuation of δ, mostly soon after JWH-073. Alterations of δ and θ like those described, are known to have different pathological significance. Physiologically, δ is the index of sleep depth, associated with deep stages 3 and 4 of NREM. The δ has the highest amplitude among brainwaves, with a frequency range from 0.01 to 0.1 Hz (Infra Slow Oscillations - ISO) and 0.5–4 Hz (δ). Cognitive performance and pathological condition both modulate δ and ISO. Generated mainly in the thalamus, spreads to the hippocampus and neocortex. δ increase inhibits GABA transmission (63) and T-type calcium channels (64) impacting multiple brain functions. A wide array of brain disorders involves δ alterations (65) like schizophrenia, with loss of θ pattern with δ burst oscillation, as recorded in our experiment, followed by thalamocortical dysrhythmia. Driving in turn hippocampus and cortex (64), yielded psychotic-like effects as a consequence of described SCBs intoxication (66), by mean of inhibitory effect on excitatory synaptic transmission (29, 35, 67). After JWH-073, we recorded δ bursts, overcoming other EEG bands similarly to a condition known as “δ coma”, a form of non-convulsive status epilepticus with high amplitude, rhythmic δ activity (68) or arising the “extreme delta brush” disorder (69). The δ band was predominant for hours, conditions corresponding to deep drowsiness, comparable with “drug blackout”, interspersed by brief hyper-reactivity or schizoid burst, observed at higher doses of SCBs (70, 71). This artificial “δ coma” is transitory and episodic, but in clinical prognosis, it heralds several months apart for damages ranging from severe memory impairment to generalized poor functional outcome. This cannot be underrated knowing that for a single mild dose, EEG alterations last almost 24 h after, so chronic sub-toxic abuse is likely long-term consequential deficits. Different δ changes between the hippocampus and cortex are probably in consequence of the inhibitory activity of JWH-073 and/or of different connections with the thalamus. The previously described θ/ δ unbalance may produce a peculiar area-dependent alteration, with hypersynchrony in the cortex and a shift of synchrony toward a loss of functionality in the hippocampus (61). Alteration of cortical θ/δ with hypersynchrony may reproduce loss of consciousness during sedation (72). In the hippocampus, the altered θ/δ ratio likely reflects *in vivo* NOR test outcome matching LTP failure for *in vitro* results. “Hippocampal θ rhythm” is a known physiological pattern crucial for memory and navigation (73). Hippocampal θ rhythm depends on projections on the medial septal area from the hypothalamus and brainstem, working as a relay for hippocampal CA3 neurons and involved in reward systems. Alterations of hippocampal θ and γ are markers of psychotic crises, switching from synchronized to chaotic. Neural synchronization regard functions like attention, memory, and sensory-motor integration and behavior. Acute smoked THC has been demonstrated capable of reproducing this loss of synchronism concurrently with psychotic alterations and loss of functionality, like above described (61), while changes have been noticed only for γ (74) and not caring about θ. Cannabinoid control *via* the endocannabinoid system of hippocampal functions is tightly correlated with control of network hippocampal oscillations, both for memory formation mechanisms and in the coding of spatial\non-spatial information (75). Rhythm disruption likely reflects the synaptic inhibition of glutamate and GABA (53, 67), with differential modulation after uneven CB1R area density. JWH-073 effect, combined a δ prevailing in the cortex with γ and θ depression in the hippocampus. This heralds a complex scenario of persistent deep cognitive depression, involving at the same time: the drive to exploration and knowledge (cortex), with the short-memory process (hippocampus). Being recognition-perception activity blurred (high δ) and hippocampal event storage failing (depressed γ and θ), the necessity of matching these to recreate a reality perception fails to de-cluttering the process information for a functional decision-making activity (76). Immobility observed could be a loss of drive to explore (messed up reality) and it seems to be associated with sensory impairment (5, 38) than drug interference with motor coordination. Spotting for epileptic traces from intersecting EEG data with simultaneous behavior, i.e., motor activity, converges to a temporary blackout than freezing or catalepsy for the seizure of index 1 or 2 (36).

#### Translation to human toxicology

Considering the remarkable fast change seen in spectrograms (Figures 4, 8), alterations are dramatic soon after 1 min from the administration. Smoking in humans as a way of administration worsens the negative effects of a sudden and massive EEG alteration. Therefore, chronic smoked mild doses of JWH-073, most likely result in cumulative toxicity and permanent subtle alterations overall for adolescent exposure (77). Indeed, differently from the well-known JWH-018, no fatal intoxication has been attributed to JWH-073 (78). The absence of epileptic events contributes to the perception of JWH-073 for safer abuse. In fact, the observed “spikes” in the cortex and hippocampus, originate from δ exacerbation, labeled as “δ epilepsy”, involving almost exclusively δ band while typical of epileptic activity involves interictal faster frequencies domain (79).

## Conclusion

Taken together these data depict a profile for JWH-073 as SCB capable at a sub-toxic dose (5) to induce immediate and long-lasting behavioral, mnemonic, and electrophysiological alterations. Consequential impairments like perceptive blackout, schizoid traits, non-convulsive epileptic bursts, and inconstant memory effectiveness may lead to hazardous compromising of demanding psychomotor activity (e.g., driving) (47). Thus, further attention will be required on the diffusion of SCs such as JWH-073 that are mistakenly perceived as “safe drugs” (80).

## Data availability statement

The original contributions presented in the study are included in the article/Supplementary material, further inquiries can be directed to the corresponding author.

## Ethics statement

Experimental protocols were approved by the Italian Ministry of Health (license n. 335/2016-PR and license n. 956/2020-PR) and by the Animal Welfare Body of the University of Ferrara.

## Author contributions

MB and MM contributed to the conception and design of the study. MT, SB, RA, GC, BM, MS, and VC performed *in vivo* experiments. MB performed *in vitro* and *in vivo* electrophysiological studies. MB and MM wrote the manuscript. MT, SB, RA, GC, BM, and GS edited sections of the manuscript. MB, MT, SB, and VC performed statistical analysis. All authors contributed to the manuscript revision, and read and approved the submitted version.

## Funding

This research has been funded by the Drug Policies Department, Presidency of the Council of Ministers, Italy (project: Effects of NPS: development of a multicentric research for the information enhancement of the Early Warning System to MM), by local funds from the University of Ferrara (FAR 2020 and FAR 2021 to MM).

## Conflict of interest

The authors declare that the research was conducted in the absence of any commercial or financial relationships that could be construed as a potential conflict of interest.

## Publisher's note

All claims expressed in this article are solely those of the authors and do not necessarily represent those of their affiliated organizations, or those of the publisher, the editors and the reviewers. Any product that may be evaluated in this article, or claim that may be made by its manufacturer, is not guaranteed or endorsed by the publisher.
