# Supplementary material for: Synthetic cannabinoid JWH-073 alters both acute behavior and in vivo/vitro electrophysiological responses in mice
Source: Front Psychiatry. 2022 Oct 21;13:953909. doi: 10.3389/fpsyt.2022.953909 (PMC9634257; doi:10.3389/fpsyt.2022.953909)
Supplement: Supplementary file 1 [file Data_Sheet_1.docx]

**Supplementary materials**

In our previous study (Ossato et al., 2016) we investigated the in vivo pharmaco-toxicological activity of JWH-073 (exploring a broad range of doses: 0.01, 0.1, 1, 3, 6 and 15 mg/kg) by using a battery of behavioral tests widely used in studies of “safety-pharmacology” for the preclinical characterization of new molecules in rodents. In vivo results showed that JWH-073, dose-dependently induced body hypothermia, increased pain threshold to both noxious mechanical and thermal stimuli, caused catalepsy, reduced motor activity, impaired sensorimotor responses (visual, acoustic and tactile), caused seizures, myoclonia, hyperreflexia and promote aggressiveness in mice. Moreover, microdialysis study in freely moving mice showed that systemic administration of JWH-073 stimulated dopamine release in the nucleus accumbens. Behavioral, neurological and neurochemical effects induced by JWH-073 at 1 and 6 mg/kg were fully prevented by the selective CB1 receptor antagonist/inverse agonist AM251. In particular, the JWH-073 at 1 mg/kg is the dose that did not induce severe neurological effects (convulsions, hyperflexia, myoclonus) and physiological and neurobehavioral impairments, but caused only a transient visual object impairment and caused, more effectively than other doses, an in vivo facilitation of dopamine release in brain circuits of rewarding. Conversely, higher dosages (3 to15 mg/kg) of JWH-073 induced several toxic effects such as seizures, myoclonia, hyperreflexia and aggressiveness in mice. Therefore, on the basis of previous pharmaco-toxicological studies (Ossato et al., 2016), we defined the dose of JWH-073 at 1 mg/kg as "mild" and we decide to investigate more accurately its effects by the in vivo EEG technique in awake mice associated with behavioral observations. In fact, this dose, transiently impairing the visual sensorimotor response (possible visual sensory distortions) and facilitating the release of dopamine, appears to be, from a translational point of view, the most representative of a dosage considered safe, devoid of evident toxic effects and with positive effects on reinforcement and rewarding mechanisms.

To investigate whether 1 mg/kg JWH-073 affected the sensorimotor (visual, acoustic and tactile) and motor (bar test and spontaneous motor activity) responses in mice, we used a battery of "safety-pharmacology" behavioral tests. Functional observational tests were carried out in a consecutive manner according to the following time scheme: 1) detection of spontaneous motor activity, 2) observation of visual object (frontal and lateral view), 3) acoustic response, 4) overall tactile (vibrissae, corneal and pinnae reflexes) responses and 5) bar test for evaluation of the akinetic/cataleptic state of mice. Tests were measured at -10 (control value), 10, 25, 105, 120 minutes and 24 hours after vehicle or JWH-073 injections. At 6) Novel Object Recognition test that was measured at 120 minutes and 24 hours after vehicle or JWH-073 injections.

1) Detection of spontaneous motor activity.

Alterations of spontaneous motor activity induced by JWH-073 was measured in mice undergoing EEG recording by using the ANY-maze video-tracking system (Ugo Basile, application version 4.99g Beta). The parameter measured was the time of movement (sec; the amount of time in seconds the animal was moving during the test) that it was analysed every 5 min in each time point (-10, 10, 25, 105, 120 minutes and 24 hours post injection). Spontaneous motor activity was recorder by using a camera (B/W USB Camera day&night with varifocal lens; Ugo Basile, Italy) and movies were analyzed off-line by a trained operator who did not know the drug treatments performed (Marti et al., 2019). In order to evaluate to analyze and correlate in more detail the degree of motor activity (potential sudden and temporary motor blocks) with the EEG activity traces, the horizontal locomotion of the mouse was monitored and analyzed every 5 min for 1 hour (Figure X).

2) Observation of visual object (frontal and lateral view)

Visual object response test was used to evaluate the ability of the mouse to see an object approaching from the front (frontal view) or the side (lateral view) that typically induces the animal to shift or turn the head, bring the forelimbs in the position of "defense" or retreat from it. For the frontal visual response, a white horizontal bar was moved frontally to the mouse head and the manoeuvre was repeated 3 times. For the lateral visual response, a small dentist’s mirror was moved into the mouse’s field of view in a horizontal arc, until the stimulus was between the mouse’s eyes. The procedure was conducted bilaterally (Ossato et al., 2015, 2018) and was repeated 3 times. The score assigned was 1 if there was a reflection in the mouse movement or 0 if it was not present. The total value was calculated by adding the scores obtained in the frontal with those obtained in the lateral visual object response test (overall score: 9).

3) Evaluation of acoustic response

Acoustic response measures the reflex of the mouse in response to an acoustic stimulus produced behind the animal (Ossato et al., 2015, 2018; Bilel et al., 2020). In particular, four acoustic stimuli of different intensity and frequency were tested. A snap of the fingers (four snaps repeated in 1.5 sec), a sharp click (produced by a metal instrument; four clicks repeated in 1.5 sec), an acute sound (produced by an audiometer; frequency: 5.0-5.1 kHz) and a severe sound (produced by an audiometer; frequency: 125-150 Hz). Each test was repeated 3 times. The score assigned was 1 if there was a response or 0 if it was not present, for a total score of 3 for each sound. The acoustic total score was calculated by adding the scores obtained in the four tests (overall score: 12). The background noise (about 40 ± 4 dB) and the sound from the instruments were measured with a digital sound level meter.

4) Evaluation of overall tactile response

Tactile response in the mouse was verified through vibrissae, corneal and pinnae reflexes (Ossato et al., 2016, 2018; Bilel et al., 2020). Data is expressed as the sum of the three above-mentioned parameters. Vibrissae reflex was evaluated by touching vibrissae (right and left) with a thin hypodermic needle once for side giving a value of 1 if there was a reflex (turning of the head to the side of touch or vibrissae movement) or 0 if not present (overall score: 2). Corneal reflex was assessed by gently touching the cornea of the mouse with a thin hypodermic needle and evaluating the response: the score assigned was 1 if the mouse moved only the head, 2 if it only closed the eyelid, 3 if it closed the lid and moved the head. The procedure was conducted bilaterally (overall score: 6). Pinna reflex was assessed by touching pavilions (left and right) with a thin hypodermic needle: first the interior pavilions and then the external. This test was repeated twice for side giving a score of 1 if a reflex was present and 0 if it was not present (overall score: 4).

5) Evaluation of catalepsy (bar test).

In the bar test, the mouse’s forelimbs were placed on a plastic bar (height 6 cm). The time spent on the bar was measured (immobility cut off: 20 s), and akinesia was calculated as the total time spent on the bar after three consecutive trials (total maximal time of catalepsy: 60 s; Canazza et al., 2016; Bilel et al., 2020b).

6) Novel Object Recognition test

Exploration time of familiar (A) and novel (B) objects was detected. The novel object preference was quantified as Recognition Index (RI) calculated as: (novel B - familiar A) / (novel B + familiar A). Using this metric, scores approaching zero reflect no preference (impairment of recognition memory), positive values reflect preference for the novel object (good recognition memory) while negative numbers reflect preference for the familiar (impairment of recognition memory). Moreover, the Total Object Exploration (TOE) time (sec) spent by the animal in the choice phase at 2 hrs (familiar A + novel B) and 24 hrs (familiar A + novel C) was calculated to investigate the effect of drugs on spontaneous object exploration (Barbieri et al., 2016). Animals that spent less than 10 s exploring both objects were excluded from the study and replaced by other animals.
